# Vitamin C and Its Role in Periodontal Diseases – The Past and the Present: A Narrative Review

**DOI:** 10.3290/j.ohpd.a44306

**Published:** 2020-04-01

**Authors:** Ubele Van der Velden

**Affiliations:** a Emeritus Professor, Department of Periodontology, Academic Centre for Dentistry Amsterdam (ACTA), University of Amsterdam and VU University, Amsterdam, The Netherlands. Study design, literature collection, analysis and writing the manuscript.

**Keywords:** ascorbic acid, gingivitis, periodontitis, scurvy, vitamin C

## Abstract

**Summary:** In humans, ascorbic acid, better known as vitamin C, is a true vitamin because humans lack the ability to synthesise it. Vitamin C exhibits a number of enzymatic and non-enzymatic effects but all are accounted for by the ability of vitamin C to donate electrons and therefore acts as a reducing agent. It has a wide range of functions. For example, it acts as co-factor for a number of enzymes including those involved in collagen hydroxylation, prevents oxidative damage to DNA and intracellular proteins, and in plasma it increases endothelium-dependent vasodilatation, and reduces extracellular oxidants from neutrophils. Deficiency in vitamin C results in the potentially fatal disease scurvy, which can be cured only by administering vitamin C. It has been shown that in individuals with gingivitis and periodontitis, plasma vitamin C levels are lower than in healthy controls. In periodontitis, a reduced capacity to absorb vitamin C may play a role. The manner in which vitamin C data are obtained from blood significantly impacts the final value obtained and therefore data validity. Plasma vitamin C levels of 56.8 μmol/l may be regarded as the optimum plasma level. In order to achieve this level, at least 200 mg vitamin C per day should be ingested. It is advisable to obtain vitamin C through the consumption of fruit and vegetables rather than supplements.

Ascorbic acid is better known as vitamin C, and whilst 99% is present under physiological conditions as the ascorbate anion,^11^ the term vitamin C will be used throughout this paper. Vitamin C is a true vitamin because humans lack the ability to synthesise it, due to a modification of the gene encoding gulonolactone oxidase, an enzyme needed as the last step in the synthesis pathway of vitamin C.^55^ From an evolutionary perspective (Fig 1), it is interesting to note that at the bottom of the phylogenetic tree, insects, invertebrates and fishes lack the ability to synthesise vitamin C. The ability to synthesise vitamin C started in the kidneys of amphibians and was maintained in reptiles, but transferred to the liver in mammals. Remarkably, the capacity to synthesise vitamin C disappeared in guinea pigs, bats (flying mammals), capybaras, monkeys and humans,^18,20^ and also in some birds.^19^

**Fig 1:**
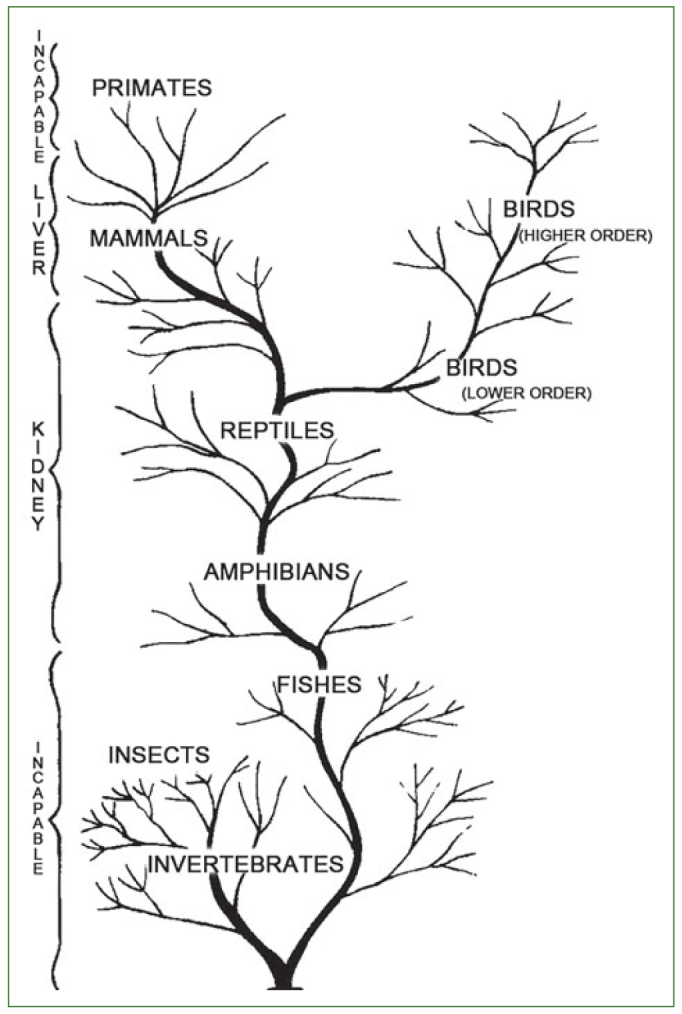
Schematic representation of vitamin C synthesising abilities of various species of animals in relation to their phylogeny (from Chatterjee^18^).

## Scurvy

Deficiency in vitamin C results in the potentially fatal disease scurvy, which can be cured only by administering vitamin C.^57^ Scurvy is probably one of the oldest diseases in human history, with the first reports documented in the Ebers Papyrus, an Egyptian medical papyrus of herbal knowledge dating to circa 1550 BC. This document described not only the diagnosis, but also treatment by means of eating onions and vegetables, both of which are rich in vitamin C. The first clear description of the disease is attributed to Hippocrates (460 BC–370 BC) but the disease was also mentioned by several Roman authors of the ancient world.^51^

Vasco da Gama, a maritime explorer, sailed from Lisbon on July 9, 1497 with about 140 Portuguese sailors and reached the southeastern coast of Africa^7^ months later. The records of the journey indicated ‘many of our men fell ill here, their feet and hands swelling and their gums grown over their teeth so that they could not eat.’ On April 6,1498 came the opportunity to purchase oranges from Moorish traders. Just 6 days later it is stated ‘all our sick recovered their health’.^10^ Decades later the Dutchman Jan van Riebeeck founded the fortified intermediate station ‘Good Hope’ (future Cape Town) in 1652 for the ‘VOC’ (Verenigde Oost Indische Compagnie) trade route between the Netherlands and the East Indies. The primary purpose of this way station was to provide fresh water, vegetables, and meat for the VOC fleets sailing between the Dutch Republic and Batavia, as mortality rates on the journey were very high.^75^ However, the need for fresh fruit and vegetables to prevent scurvy was not recognised at the time, and as a result the disease killed many sailors between 1500-1800 AD. In perhaps the first controlled clinical trial (1753), James Lind, an officer of the British Royal Navy, selected 12 patients with scurvy, who were as similar as possible in other demographic features. He introduced 6 different diets (including sea water as a control) to each of 6 pairs of patients, and was able to conclude that eating two oranges and one lemon a day was the most effective remedy to cure scurvy: within 6 days a patient was fit for duty.^45^ James Cook, an English explorer, followed a strategy to use every opportunity to replenish the food supplies of his ships with green vegetables, sweet potatoes and coconuts.^51^ Nevertheless, up to the 20th century, scurvy claimed many victims due to a lack of understanding of the pathophysiology of vitamin C deficiency. An important scientific advancement in understanding the aetiology of scurvy was made by Holst and Frölich.^34^ Following an extensive study in guinea-pigs they were able to conclude that (quote): ‘scurvy originates in guinea-pigs as well as in man as a result of a diet confined to some special nutriments and that at least one of these nutriments loses a deal but not all of its preventive power when boiled for half an hour at 110°C’. In 1927, several decades later, Szent-Györgyi isolated hexuronic acid, as he called it, which appeared to be the antiscorbutic factor ascorbic acid, as determined in 1932 by both Svirbely and Szent-Györgyi^70^ and King and Waugh.^40^

**Fig 2:**
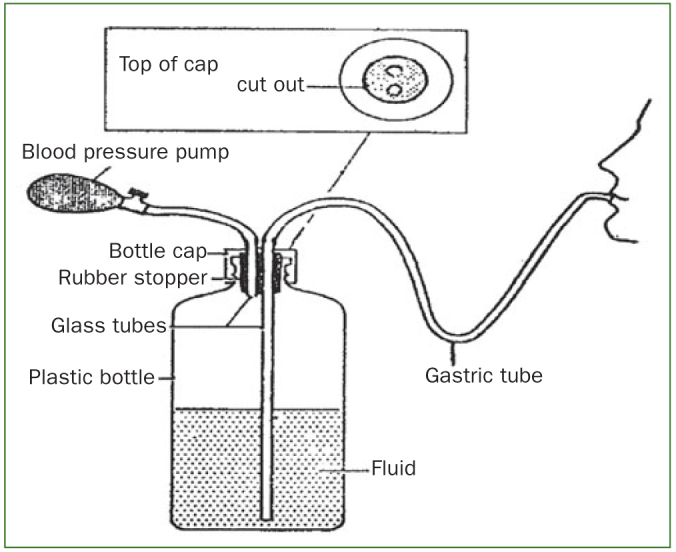
Schematic of formulated diet administration through a gastric tube by means of a pressure bottle (from Hodges et al^32^).

Over the next few years, a number of studies were undertaken and devoted to the proper assessment of the vitamin C content of human blood. Van Eekelen^74^ is believed to have conducted in 1936 the first vitamin C depletion/repletion study, in one individual. It included three episodes of 250 mg vitamin C supplementation and a period of 84 days during which a diet devoid of vitamin C was adhered to. It was concluded, based upon blood and urine analysis that the daily vitamin C dose required for adults amounts to 60 mg. It was noteworthy that at the end of the 84-day period no other symptoms of vitamin C deficiency were present, other than fatigue and irritability.^74^ Thereafter, several experimental scurvy studies were performed, of which the study by Hodges et al^32^ is most noteworthy because, in order to maximise results, the research was carried out unscrupulously and would probably not have passed a modern-day medical ethics committee. The study included 6 prisoners who volunteered to participate. They were hospitalised on a metabolic ward and given a diet totally devoid of vitamin C, but adequate in all other essential nutrients. The subjects were taught to swallow a gastric tube through which they were later administered, three times daily, a liquid formulated diet for 113 days (Fig 2). The vitamin C deficient diet was provided until convincing evidence of mild clinical scurvy appeared and until, on the 99th day, the body’s pool of vitamin C had been largely depleted. On the 100th day, repletion with vitamin C commenced using controlled levels of intake of low specific activity carbon 14-labeled vitamin C ranging from 4 to 64 mg daily. On the 113th day of the study (14th day of repletion), the subjects were changed from the liquid diet to the solid diet, which provided 2.5 mg vitamin C daily. This diet was provided until day 195, following which a final recovery period of 2 weeks was permitted using a diet of the subject’s choice, supplemented with 500 mg vitamin C. Because 2 prisoners escaped, only 4 subjects completed the study. Results showed that all 4 subjects developed clinical symptoms of scurvy: follicular hyperkeratosis of the thighs, buttocks and arms; swollen bleeding gums; perifollicular hemorrhages and congested follicles; and conjunctival hemorrhages. In addition, it was concluded that clinical symptoms of scurvy began to appear when the total body pool of vitamin C had decreased to approximately 300 mg.^32^ Once the body pool of vitamin C was replete to a level of 1500 mg, urinary loss of vitamin C began to occur.^8^ In a comparable follow-up study on 5 subjects, utilising a solid diet, Hodges et al^33^ confirmed that the total body pool of vitamin C approximates 1500 mg in healthy humans. However, more recent papers argued that the vitamin C values assessed in the reported studies likely overestimated the true amounts due to the limitations of the analytical techniques employed. Using modern HPLC electrochemical assays in a depletion/repletion study, young healthy adults attained plasma vitamin C concentrations of 8 µmol/l [1.4 mg/] without developing scurvy; however many of the subjects experienced lassitude and it was considered unsafe to further deplete them of vitamin C.^44,57^

## Biological Functions of Vitamin C

Vitamin C exhibits a number of enzymatic and non-enzymatic effects but all are accounted for by a single chemical property: vitamin C is an electron donor and thus a reducing agent. Padayatty and Levine^57^ recently documented the known and proposed actions of vitamin C. In mammals it acts as co-factor for a number of enzymes including those involved in norepinephrine biosynthesis, collagen hydroxylation, hypoxia-inducible factor (HIF) hydroxylation and regulation of HIF (HIF-1 is a transcription factor that is key to oxygen sensing), amidation of peptide hormones, carnitine biosynthesis, tyrosine metabolism and histone demethylation. As an antioxidant micronutrient, vitamin C regulates gene expression and mRNA translation via redox-sensitive gene transcription factors, and prevents oxidative damage to DNA and intracellular proteins. In plasma it increases endothelium-dependent vasodilatation, reduces extracellular oxidants from neutrophils, reduces low-density lipoprotein oxidation, quenches aqueous peroxyl radicals and lipid peroxidation products. In addition, it prevents formation of N-nitroso compounds in the stomach. Under certain conditions, however, vitamin C may be a pro-oxidant, especially when it is present in pharmacological concentrations. However, in nature, pharmacologic concentrations of vitamin C do not arise in the blood or extracellular fluids.^57^ Overall, it is plausible that vitamin C, through its antioxidant actions and the other biological mechanisms, plays a role in preventing certain cancers, enhancing immune function, and ameliorating chronic inflammatory conditions.^27^

## Vitamin C and General Health

Due to the broad and diverse biological functions of vitamin C, it seems logical that reduced availability of vitamin C will be associated with disease. Indeed, apart from scurvy, low vitamin C plasma levels have been related to diabetes,^67,79^ cardiovascular diseases^39,61^ and cancer.^37,47^ However, the presence of low plasma values should be interpreted with care, because this may not only relate to low dietary intake or reduced absorption, but also to increased metabolism of vitamin C. For example, smokers metabolise more vitamin C than non-smokers.^65^ Moreover, in a systematic review, it was shown that *Helicobacter pylori* chronic gastritis infection is associated with reduced levels of vitamin C in plasma and gastric juice. Eradication of *H. pylori* had no clear effect on plasma vitamin C levels but levels increased significantly in gastric juice, indicating that the eradication therapy yielded a significant enhancement of the vitamin C pool.^42^

Based on population studies plasma vitamin C levels have been classified as deficient (<11.3 μmol/L [<2 mg/L]*), depleted (11.4–22.6 μmol/L [2.0–3.9 mg/L]*), normal (22.7–56.7 μmol/L [4.0–9.9 mg/L]*) and saturated (≥ 56.8 μmol/L [≥ 10.0 mg/L]*) according to internationally established limits.^48^ It has been estimated that in the industrialised world, vitamin C depletion affects as many as 10% of adults.^46^ Furthermore, vitamin C-depleted men have a 57% higher risk of all-cause mortality after 12–16 years follow-up than men with plasma levels vitamin C > 74 μmol/l.^29^ The hypothesis that vitamin C can reduce cardiovascular risk is, for example, supported by the finding that vitamin C reduces monocyte adhesion to the endothelium and therefore the likelihood of atheroma formation.^78^ In addition, vitamin C has been shown to restore the endothelial nitric oxide synthase enzymatic activity, resulting in an increase of nitric oxide, a potent vasodilator.^22^

**Fig 3:**
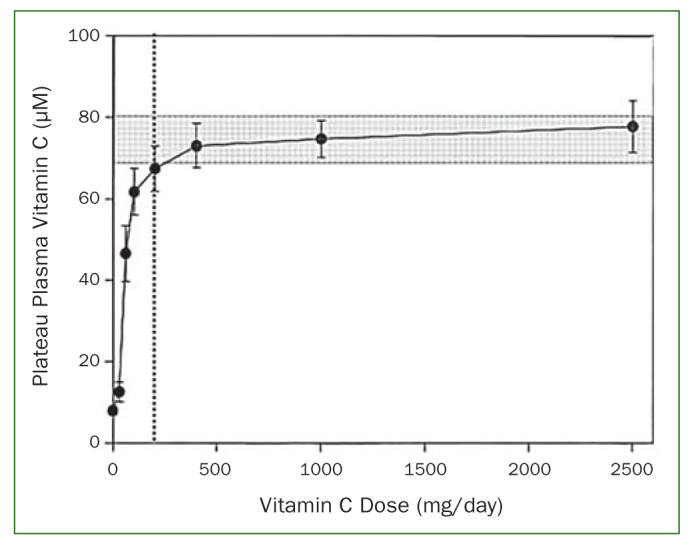
Two hundred mg of vitamin C (vertical dotted line) as optimum daily intake based on a) near-saturating plateau plasma vitamin C concentration of ≥ 70 μM (shaded area) and b) first dose beyond the steep, linear increase in plasma concentration at vitamin C intakes of 30–100 mg/day (from Levine et al^44^ and Frei et al^17^).

**Fig 4:**
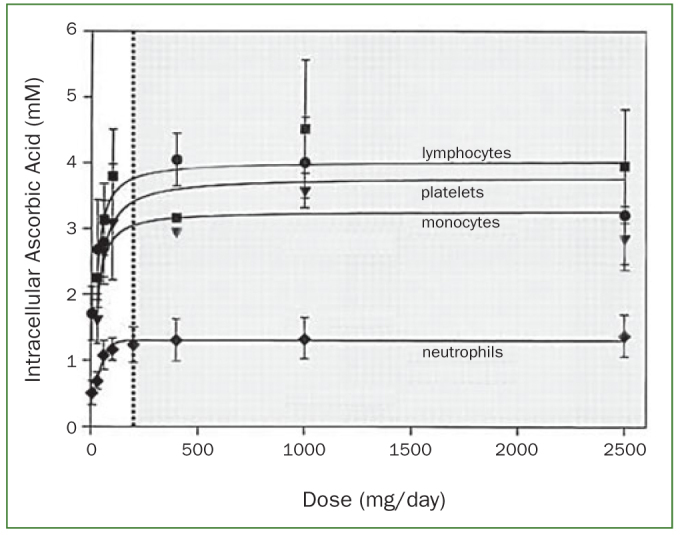
Neutrophils and other circulating cells are saturated with vitamin C at an intake level ≥ 200 mg/day (dotted line, shaded area), as indicated by intracellular ascorbic acid concentrations (from Levine et al^44^ and Frei et al^17^).

In light of the above, it is unsurprising that the optimal amount of dietary vitamin C intake has been a matter of debate. It has been shown that the minimum amount of vitamin C necessary to prevent or cure scurvy appears to be slightly less than 10 mg daily.^33^ A recommended daily vitamin C allowance (RDA) for an adult male was set by the Food and Nutrition Board of the US in 1943 at 75 mg/day, but later reduced to 60 mg/day, and in 1973 to 45 mg/day. The principal consideration in setting these values was the prevention of scurvy.^60^ However, scurvy is not the first symptom of vitamin C deficiency, more a final consequence, and a pre-mortal syndrome. There is also a very wide gap in the body’s vitamin C levels between scurvy and full health.^38^ In this context, Pauling^60^ argued that the discussion should be whether an intake of 100-250 mg/day, required for ‘tissue saturation’, is essential to normal good health. Because of such discussions, the Food and Nutrition Board of the US increased the RDA of vitamin C to 60 mg/day in 1980, which was reconfirmed in the 1989 report.^25,26^ The RDA for vitamin C of 60 mg daily is based on the threshold for urinary excretion of the vitamin and on preventing scurvy with a margin of safety. However, whether this is the optimal dose of vitamin C may still be challenged. Levine et al^44^ argued that to establish an RDA for a vitamin, it is necessary to determine vitamin concentrations in plasma and tissues in relation to vitamin dose for a wide range of doses, true bioavailability or vitamin absorption at each dose, vitamin urinary excretion at each dose, and potential toxicity. Because these data are essential for an RDA for vitamin C, the authors conducted a milestone investigation, studying vitamin C depletion-repletion pharmacokinetics in seven healthy in-patient volunteers using seven doses of vitamin C ranging from 30 to 2500 mg. Subjects started with vitamin C depletion to reduce plasma vitamin C concentrations to 0.9 – 1.9 mg/l. For repletion, daily vitamin C was provided until a plateau value was obtained in the following order: daily dose of 30, 60, 100, 200, 400, 1000 and 2500 mg. Results demonstrated a steep increase in plasma steady-state concentrations of vitamin C from about 10 μmol/l at an intake of 30 mg/day to about 60 μmol/l at 100 mg/day. Vitamin C plasma concentrations were near saturation at intakes of 200–400 mg/day and reached a plateau of about 80 μmol/l at intakes of 1000 and 2500 mg/day (Fig 3). Hence, 200 mg/day is the lowest dose of vitamin C to approximately reach the plateau level, achieving a plasma concentration of about 70 μmol/l. The study also demonstrated that at a daily intake of 200 mg vitamin C, the lymphocytes, platelets, monocytes and neutrophils were saturated with vitamin C (Fig 4). It has also been shown that at plasma vitamin C levels of 56.8 μmol/l, renal excretion of vitamin C starts to increase and that at values higher than 70 μmol/l renal vitamin C excretion accelerates rapidly.^31^ This implies that plasma vitamin C levels of 56.8 μmol/l may be regarded as to the optimal vitamin C plasma level.

Another interesting aspect of the study by Levine et al^44^ was the relatively large individual variation in steady-state plasma concentrations of vitamin C already present in the small group of 7 participants. This was evident especially for the 60-mg vitamin C dose, with the lowest participant reaching a level of 14.9 μmol/l (still severely depleted), whilst the highest reached a concentration of 58.8 μmol/l (saturated). Such individual variation was also reported in a recent vitamin C supplementation study in a group of 98 Indonesian tea workers with poor dietary conditions.^3^ In this study, in which no relationship existed between baseline plasma vitamin C levels and smoking status, participants consumed one vitamin C tablet per day under supervision, containing 200 mg Ester C calcium ascorbate, 25 mg calcium threonate and 100 mg citrus flavonoids for three months. Results showed that after three months of supplementation 73.5% of this population achieved fasting plasma vitamin C levels ≥ 56.8 μmol/l (optimal values). In the remaining participants, plasma vitamin C values varied from 46.8–56.8 μmol/l in 12.3%, 36.8–46.8 μmol/l in 11.2%, and 22.7 – 36.8 μmol/l in 3%, implying that probably 14% of this population have difficulties in absorbing vitamin C. Differences between participants are likely due to genetic variations in the vitamin C transporter protein SVCT1, which has been shown to influence plasma vitamin C concentrations.^13^

## Vitamin C and Gingival Diseases

As previously discussed, vitamin C plays a clear aetiological role in oral manifestations of scurvy, although it is less clear to what extent it is involved in other periodontal diseases. In the past, vitamin C deficiency has been implicated in necrotising ulcerative gingivitis (NUG).^14^ NUG is a specific form of periodontal disease, characterised by ‘punched out’ ulcerated papillae, gingival bleeding and pain. Primary risk indicators for the disease include smoking, psychological stress, malnutrition, immunosuppression and pre-existing gingivitis.^62^ The most extensive study to investigate a potential role for vitamin C depletion in NUG was a case control study including 60 NUG patients.^53^ Each case was matched by age, race and sex to one control who was a current friend or when this was not possible a University employee. Controls had to have no history of NUG. Dietary vitamin C intake was estimated by the total number of servings of fresh fruits, juices and/or vegetables consumed the previous day and plasma levels of vitamin C were also assayed. Results indicated that NUG cases ingested nearly 60% fewer servings containing vitamin C during the preceding day than controls and that plasma vitamin C levels were lower in the cases than controls. However, these differences were no longer statistically significant after adjusting for social class. It is important to note that the results were based on assessments obtained two or more months after the initial lesions healed. Furthermore, the plasma vitamin C levels for cases and controls were 70 and 100 μmol/l, respectively. Although these values may be an overestimation of the true vitamin C levels due to the analytical method employed to determine vitamin C at that time, it is unlikely that the reported cases were vitamin C depleted. This implies that the risk indicators of smoking, psychological stress, malnutrition, immunosuppression, low social class and pre-existing gingivitis are far more important for the development of NUG than vitamin C as such.

Understanding the relationship between gingivitis and vitamin C is hampered by the difficulty in distinguishing gingivitis from periodontitis, because attachment loss measurements are seldom included in gingivitis studies. Nevertheless, whilst there is little evidence from well-defined gingivitis populations, it may be suggested that in individuals with gingivitis:

- plasma vitamin C levels are lower than in healthy controls^28^
- the degree of gingival inflammation is directly related to plasma vitamin C levels^43^
- increased vitamin C intake, either by diet or supplements, can reduce gingival bleeding.^28,36,43^

It is interesting to note that the experimental gingivitis model was employed in only two studies to investigate the effect of vitamin C supplementation on the development of gingival inflammation. The effect of megadoses vitamin C supplementation (daily dose of 1500 mg vitamin C) was studied in a four-week experimental gingivitis model including 24 dental students (12 test and 12 controls). Results showed no difference in the amount of gingivitis after four weeks between test and control volunteers.^76^ However, the volunteer students had a daily dietary vitamin C intake amounting to almost 200 mg prior to the study. Therefore, these volunteers were already saturated with vitamin C through their daily diet. This implies that the surplus of vitamin C is efficiently excreted and hence it is highly unlikely that the test group benefitted from further vitamin C supplementation.^50^ The second experimental gingivitis study included three groups of 16 non-smoking dental students supplemented daily with either 200 g guava fruit (containing 260 mg vitamin C), 200 mg synthetic vitamin C or water for four weeks. In a pre-trial period of 14 days, all participants received oral hygiene instructions and prophylaxis treatment, thereafter two weeks of experimental gingivitis was initiated.^4^ Results showed that consumption of either 200 g guava/day or 200 mg synthetic vitamin C/day, prior to and during the oral hygiene abstention period, has a strong preventive effect on the development of experimental gingivitis as compared to the control group that developed the usual amount of experimental gingivitis. In view of the above literature, it can be concluded that an inverse relationship exists between vitamin C and gingivitis. In addition, no benefit of vitamin C supplementation can be anticipated in individuals already ingesting optimal amounts of vitamin C (200 mg/day), although this dose may be higher in smokers and in individuals that have difficulties in absorbing vitamin C.

## Vitamin C and Periodontitis

In early epidemiological studies, no relationship could be demonstrated between plasma vitamin C levels and the periodontal status.^9,23,63,65^ This may be attributed to the use of Russell’s Periodontal Index for the periodontal evaluation, the relatively large number of younger individuals with gingivitis only, blood sampling/laboratory procedures, and/or lack of control of confounding factors. It is noteworthy, based on the NHANES I study, that Ismail et al^35^ found a weak but significant negative correlation between dietary vitamin C intake and Russell’s Periodontal Index after controlling for the potentially confounding variables of age, sex, race, education, income and oral hygiene status. In addition, more recent studies have found a relationship between vitamin C and periodontitis. Vogel and Wechsler^77^ showed that the daily intake of vitamin C in a group of periodontitis patients was significantly less than in the control subjects. Blignaut and Grobler^12^ compared the periodontal condition of workers in citrus fruit-producing farms to that of workers in grain-producing farms. The only significant difference in the diets of the groups was the large amounts of fresh fruit consumed by the fruit-farm workers who had free access to the fruit produced on the farms. Results showed that deeper pockets (CPITN code 3 and 4) occurred far less frequently in subjects who consumed citrus fruit. Using the NHANES III survey, Nishida et al^54^ found that the dietary intake of vitamin C showed a weak, but statistically significant inverse relationship with periodontal disease in current and former smokers. Smokers with the lowest intake of vitamin C were likely to have the worst periodontal condition. Using the same NHANES III data set, Chapple et al^16^ found a strong and consistent inverse association between serum vitamin C concentrations and the prevalence of periodontitis in adjusted models (multiple logistic regression analysis adjusted for age, gender, race/ethnicity, BMI, cigarette smoking, oral contraceptives and hormone replacement therapy use, diabetes, poverty income ratio, and education). Their results also showed stronger inverse associations between serum vitamin C concentrations and periodontitis among a subgroup of never-smokers than within the full sample. Amarasena et al^6^ showed an inverse relationship between serum vitamin C levels and attachment loss irrespective of smoking, diabetes, oral hygiene, sex or number of teeth present in an elderly population of community dwelling Japanese subjects. In an Indonesian population deprived of dental care and a diet low in vitamin C, a significant inverse relationship between plasma vitamin C levels and attachment loss was observed.^5^ In a nationally representative sample of young adult Koreans, periodontitis was associated with a lower intakes of vitamin C^59^ (multivariate logistic regression analyses adjusted for gender, age, household income, education level, diabetes, obesity, oral health behaviours and number of decayed permanent teeth). In addition, the most recent analysis of the NHANES data collected in 2011-2014 also demonstrated that the lower the vitamin C intake, the more severe the periodontal condition^49^ (complex samples general linear model adjusted for age, gender, race, education level, family income, diabetes history and hypertension history).

Case-control studies have shown that periodontitis patients have lower plasma vitamin C levels than the healthy controls.^7,41,58,68,69,72^ Also, periodontitis patients who smoke appear to have lower plasma vitamin C levels than non-smoker patients.^58,68^ Based on a 7-day diet record, Staudte et al^69^ also showed that periodontitis patients had significantly lower rates of vitamin C intake compared to controls. It is interesting to note that in the study by Kuzmanova et al,^41^ in which controls were matched for age, sex, race and smoking habit, the periodontally healthy control group showed a significant positive correlation between plasma vitamin C levels and the total amount of vitamin C ingested during the 3 days prior to blood sampling. In contrast, such a relationship was absent in the periodontitis patient group although there was no significant difference in the amount of dietary vitamin C intake between patients and controls. This finding may relate to a higher number of individuals in the periodontitis group who may have difficulties in absorbing vitamin C compared to the control group. As previously discussed, these inter-individual differences may be due to genetic variations in the vitamin C transporter protein SVCT1 that has been shown to influence plasma vitamin C concentrations.^13^ Moreover, a recent study suggested that genetic variance in SLC23A1 encoding for SVCT1 was related to periodontitis.^21^

Low plasma vitamin C values may arise due to low dietary intake or reduced absorption, but also due to increased metabolism of vitamin C. The finding that a non-surgical treatment of periodontitis resulted in a significant increase in plasma vitamin C levels from 22.7 μmol/l to 28.4 μmol/l^7^ supports the latter mechanism. In a non-surgical intervention study, carried out over two appointments separated by one week, the test group received 450 mg vitamin C supplementation daily for two weeks. Two weeks following treatment, there was no additional improvement in terms of bleeding on probing and probing pocket depth.^28^ In another non-surgical treatment study carried out in a 48-h period, an adjunctive dose of 2000 mg vitamin C for 4 weeks was employed. Following periodontal treatment, the plasma total antioxidant capacity (TAOC) in periodontitis patients had increased to the level of the healthy controls. However, no differences in clinical improvement or in TAOC values could be seen between the periodontitis patients with and without the supplements. Unfortunately, no plasma vitamin C levels were reported.^1^ Moreover, the use of such TAOC assays has been criticised, since with some notable exceptions (e.g. Maxwell et al^52^), the target antioxidant species for the majority of these assays have not been determined,^66^ and the measures they record thus represent an unknown. In dietary supplementation studies of periodontitis patients, it has been shown that vitamin C supplementation by means of fruits as sole treatment modality, results in a significant reduction of bleeding on probing. This effect was shown in a study using 2 grapefruits of 300 g per day providing about 180 mg vitamin C^68^ and another randomised controlled clinical trial where patients in the experimental group consumed 2 kiwifruits per day providing between 100-200 mg vitamin C.^30^ In the latter study, in addition to a reduction in bleeding on probing, a reduction in plaque scores was also found. However, no supplementation effect for the subsequently performed non-surgical periodontal therapy could be analysed. By contrast, a dietary supplementation study with fruit/vegetable/berry juice powder concentrates (including 200 mg vitamin C per day), simultaneously with 4-week, quadrant-wise non-surgical periodontal therapy, showed additional initial pocket depth reductions (at 2 months after treatment) and subsequent additional improvements in bleeding on probing (at 5 months) and plaque scores (at 8 months).^17^ Other intervention studies combining micronutrients (vitamins and minerals) and phytonutrients (chemical compounds produced by plants) are beyond the scope of this review. Taking into account that in the absence of periodontal treatment, vitamin C supplementation reduces bleeding on probing in periodontitis patients, but that non-surgical treatment is very effective in terms of pocket depth reduction, gains in clinical attachment and reduced bleeding on probing,^73^ an additional supplementation effect may be difficult to demonstrate, unless volunteers are vitamin C deficient.

## Blood Sampling and Vitamin C Assessment

The manner in which vitamin C data are obtained from blood impacts significantly on the final value obtained and therefore upon data validity. In epidemiological studies, vitamin C assessments have been performed both in serum and plasma. Firstly, assessment in plasma is preferred, since in this procedure, the sample is in contact with oxygen as briefly as possible. For serum, blood is first allowed to clot by leaving it undisturbed at room temperature usually for 15-30 minutes, during which vitamin C oxidation and vitamin C uptake by blood cells can occur. In the past, in those studies that found no relationship between plasma/serum vitamin C levels and periodontal status, blood handling procedures may have been different. For example, Barros and Witkop^9^ write: ‘blood samples, collected daily in the field, were placed in an ice container and flown to laboratories in Santiago. Nearly all samples were analysed within 48 h after collection’. Secondly, fruit containing vitamin C ingested some time prior to blood sampling will influence the plasma values. Levine et al^44^ showed that plasma vitamin C values peak 2-5 h after vitamin C ingestion and are back to baseline after 12 h. Consequently, fasting blood samples will reflect the body pool of vitamin C taking into account that it is subject to change, as some of the more labile body pools of vitamin C may enlarge or decrease depending upon vitamin C intake.^56^ Taken together, this implies that plasma vitamin C values of non-fasting blood samples is a combination of the steady-state plasma vitamin C and the recently absorbed vitamin C, which may be less in the case of periodontitis patients. These variables play a minor role in case-control studies of sufficient size because the vitamin C assessment procedure is the same in both groups. However, they may play a role in epidemiological studies. In the aforementioned studies that found a significant inverse relationship between plasma vitamin C levels and the severity of periodontitis, it was not mentioned whether fasting or non-fasting blood samples were used.^5,6,16,58,72^ It is interesting to note that in the Indonesian population study in 2005, a significant inverse relationship between plasma vitamin C levels and attachment loss was found,^5^ whereas in a subsequent study of the same population in 2011, no relationship was found.^2^ However, applying the plasma vitamin C data of 2005 to the amount of periodontal breakdown as assed in 2011, again a significant relationship was present; but applying the plasma vitamin C data of 2011 to the attachment loss data of 2005 no relationship was found. The difference between the two studies was that in 2005, non-fasting blood samples were taken, whereas in 2011, fasting blood samples were obtained. As far as can be determined, only one other epidemiological study mentioned that fasting blood samples were employed.^23^ In the latter study, no relationship could be found between plasma vitamin C levels and the periodontal condition. As previously discussed, some periodontitis patients may be less able to take up vitamin C from ingested food than periodontally healthy controls.^41^ When using non-fasting blood samples, the previously consumed vitamin C containing food will increase the steady-state vitamin C plasma level. This increase will likely be influenced by the vitamin C absorption capacity of the individual, among other things. This implies that if present, a difference in plasma vitamin C values between periodontitis patients and healthy controls may be observed, whereas the use of fasting blood samples would mask such a relationship.

## Dietary Counselling on Vitamin C

Because vitamin C deficiency may play a role in the development and progression of periodontitis, a recent workshop concluded that dietary counselling on vitamin C intake should be undertaken.^15^ This implies that at least evaluation of the vitamin C content of the diet should become a routine part of anamnesis. This can be obtained for example by means of a self-administered dietary record in which the patients document what was consumed 3 days prior to the examination in terms of food/beverages containing vitamin C. For this purpose, a list with a selection of relatively frequently consumed fruits and vegetables containing a reasonable amount of vitamin C (≥ 10 mg/100 g) has been added (Table 1). This may help the clinician and/or patient to estimate their dietary vitamin C intake, but it is important to realise that a number of frequently consumed fruits are not present on that list, for example apples and bananas. In addition to the diet, attention should also be paid to the potential use of food supplements. Food supplements are concentrated sources of nutrients (i.e. minerals and vitamins) or other substances with nutritional or physiological effects that are marketed in ‘dose’ form (e.g. tablets, capsules, liquids in measured doses). A wide range of nutrients and other ingredients might be present in food supplements, including, but not limited to, vitamins, minerals, amino acids, essential fatty acids, fibers and various plants.^24^ Food supplements are intended to correct nutritional deficiencies which is not needed in the case of vitamin C because adequate amounts of vitamin C can easily be obtained by consuming the fruits and vegetables presented in Table 1.

**Table 1:** Vitamin C content of relatively frequently consumed fruits and vegetables (raw) containing ≥ 10 mg/100 g vitamin C^71^

Food description (raw)	mg vitamin C per 100 g	Food description (raw)	mg vitamin C per 100 g
Guava	228.3	Currents (red/white)	40.1
Black currant	181.0	Melon (cantaloupe)	36.7
Kiwi gold	161.3	Cabbage	36.6
Red bell pepper	127.7	Mango	36.4
Kale	93.4	Grapefruit	34.4
Kiwi green	92.7	Spinach	28.1
Broccoli	89.2	Blackberries	21.0
Brussels sprouts	85.0	Potato	19.7
Papaya	60.9	Melon (honeydew)	18.0
Strawberry	58.8	Zucchini (courgette)	17.9
Cabbage (red)	57.0	Cranberry	14.0
Oranges	53.2	Tomato	13.7
Lemon	53.0	Green beans	12.2
Tangerine	48.8	Pomegranate	10.3
Cauliflower	48.2	Apricot	10.0
Pineapple	47.8	Avocado	10.0

## Conclusion

For humans, vitamin C is a true vitamin which must be obtained from dietary sources, because humans lack the ability to synthesise this molecule. As discussed, plasma vitamin C levels of 56.8 μmol/l may be regarded as the optimum vitamin C plasma level. In order to achieve this level, at least 200 mg vitamin C per day should be ingested. This should become general advice to periodontitis patients. However, some patients may require even more due to possibly reduced vitamin absorption and/or smoking. It is advisable to obtain the vitamin C through the consumption of fruit and vegetables rather than supplements.

## Footnotes

* In the paper of Loria et al^48^ and previous papers mg/dL or mg/L was used, later this was replaced by μmol/L.
